# Bile acid signaling in skeletal muscle homeostasis: from molecular mechanisms to clinical applications

**DOI:** 10.3389/fendo.2025.1551100

**Published:** 2025-03-12

**Authors:** Feng Jia, Xiangliang Liu, Yahui Liu

**Affiliations:** ^1^ Department of Hepatobiliary and Pancreatic Surgery, General Surgery Center, The First Hospital of Jilin University, Changchun, China; ^2^ Cancer Center, The First Hospital of Jilin University, Changchun, China

**Keywords:** bile acids, skeletal muscle, FXR, tgr5, sarcopenia, biomarkers, therapeutic targets

## Abstract

The intricate relationship between bile acid metabolism and skeletal muscle function has emerged as a crucial area of research in metabolic health. This review synthesizes current evidence highlighting the fundamental role of bile acids as key signaling molecules in muscle homeostasis and their therapeutic potential in muscle-related disorders. Recent advances in molecular biology and metabolomics have revealed that bile acids, beyond their classical role in lipid absorption, function as essential regulators of muscle mass and function through multiple signaling pathways, particularly via the nuclear receptor FXR and membrane receptor TGR5. Clinical studies have demonstrated significant associations between altered bile acid profiles and muscle wasting conditions, while experimental evidence has elucidated the underlying mechanisms linking bile acid signaling to muscle protein synthesis, energy metabolism, and regeneration capacity. We critically examine the emerging therapeutic strategies targeting bile acid pathways, including receptor-specific agonists, microbiome modulators, and personalized interventions based on individual bile acid profiles. Additionally, we discuss novel diagnostic approaches utilizing bile acid-based biomarkers and their potential in early detection and monitoring of muscle disorders. This review also addresses current challenges in standardization and clinical translation while highlighting promising future directions in this rapidly evolving field. Understanding the bile acid-muscle axis may provide new opportunities for developing targeted therapies for age-related muscle loss and metabolic diseases.

## 1 Introduction

Sarcopenia, characterized by the progressive loss of skeletal muscle mass, strength, and function, has emerged as a significant public health concern affecting approximately 10-16% of older adults worldwide (1). This age-related muscle deterioration not only compromises physical function and independence but also substantially increases the risk of falls, fractures, and mortality in the aging population. As global demographics shift towards an increasingly aged population, understanding the molecular mechanisms underlying sarcopenia and identifying novel therapeutic targets has become imperative.

In recent years, bile acids have gained recognition far beyond their classical role in dietary lipid absorption. These versatile molecules have emerged as crucial signaling mediators that regulate diverse metabolic processes through both nuclear and membrane receptors (2). The discovery that bile acids function as signaling molecules has revolutionized our understanding of their physiological importance, particularly in metabolic homeostasis and tissue function regulation (3).

Intriguingly, emerging evidence suggests a complex interplay between bile acid metabolism and skeletal muscle health. The identification of bile acid receptors, particularly TGR5, in skeletal muscle tissue has opened new avenues for understanding muscle metabolism and function (4). Recent studies have demonstrated that bile acid signaling pathways can influence muscle mass, strength, and metabolic function, suggesting potential therapeutic implications for sarcopenia. Furthermore, the gut microbiota’s role in modulating bile acid composition and signaling adds another layer of complexity to this relationship (5).

This review aims to comprehensively examine the emerging connection between bile acid metabolism and skeletal muscle health, with a particular focus on its implications for sarcopenia. We will explore the molecular mechanisms through which bile acids influence muscle metabolism, discuss the role of gut microbiota in mediating these effects, and evaluate the therapeutic potential of targeting bile acid signaling pathways in treating age-related muscle loss. Understanding these relationships may provide novel insights into the pathophysiology of sarcopenia and identify promising therapeutic strategies for this increasingly prevalent condition.

## 2 Bile acid biology and signaling

Bile acids represent a unique class of steroid molecules characterized by a 24-carbon backbone structure derived from cholesterol. Their distinctive amphipathic nature arises from the spatial arrangement of hydrophilic α-oriented hydroxyl groups on one face and hydrophobic methyl groups on the opposite face. This structural organization is crucial for their biological functions, as the number and position of hydroxyl groups, along with conjugation status, determine their physiochemical properties and biological activities. Modern structural analyses have revealed that these modifications significantly influence receptor binding affinity and specificity, particularly for key receptors such as FXR and TGR5 (2, 3).

The human body produces two primary bile acids - cholic acid (CA) and chenodeoxycholic acid (CDCA) - through hepatic synthesis. These primary bile acids undergo conjugation with either glycine or taurine in hepatocytes, forming conjugated bile acids that exhibit enhanced solubility and reduced cytotoxicity. A crucial transformation occurs when these primary bile acids reach the intestine, where the gut microbiota modify them through three main processes: deconjugation by bile salt hydrolases, 7α-dehydroxylation, and epimerization. This bacterial transformation yields secondary bile acids, primarily deoxycholic acid (DCA) and lithocholic acid (LCA), which possess distinct physiological properties and signaling capabilities (6, 7).

**Figure 1:**
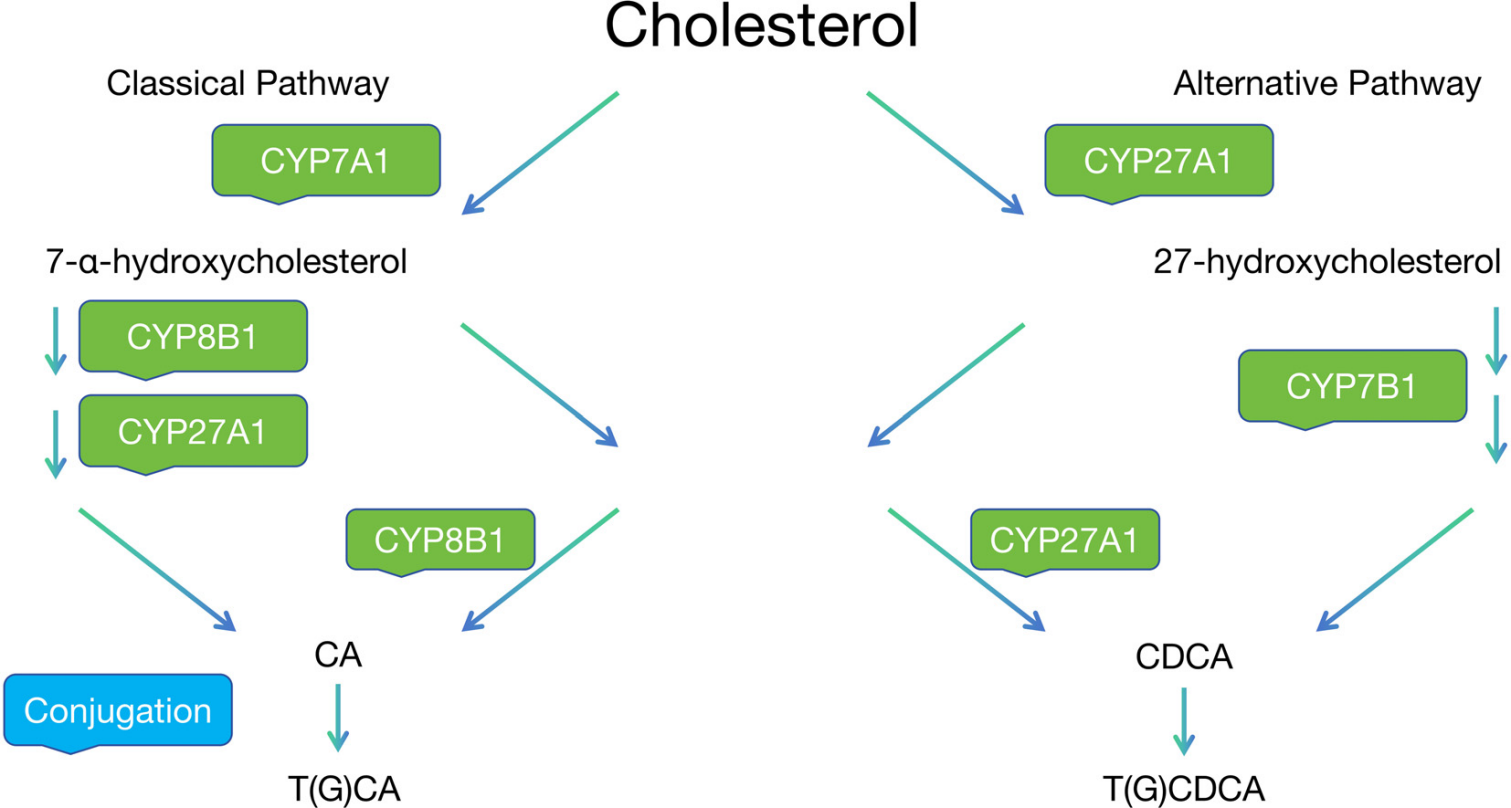
The two major pathways of bile acid synthesis.

As shown in **Figure 1**, The synthesis of bile acids occurs through two major pathways: the classical (neutral) and alternative (acidic) pathways. The classical pathway, initiated by the rate-limiting enzyme cholesterol 7α-hydroxylase (CYP7A1), accounts for approximately 90% of bile acid synthesis and is tightly regulated through sophisticated feedback mechanisms. A remarkable feature of bile acid metabolism is the enterohepatic circulation, which efficiently maintains the bile acid pool. Approximately 95% of bile acids are reabsorbed in the ileum through the apical sodium-dependent bile acid transporter (ASBT). This highly efficient recycling system involves the coordinated actions of various transporters, including NTCP in the liver and OSTα/β in the intestine, ensuring appropriate bile acid distribution while preventing cytotoxicity (8).

**Figure 2:**
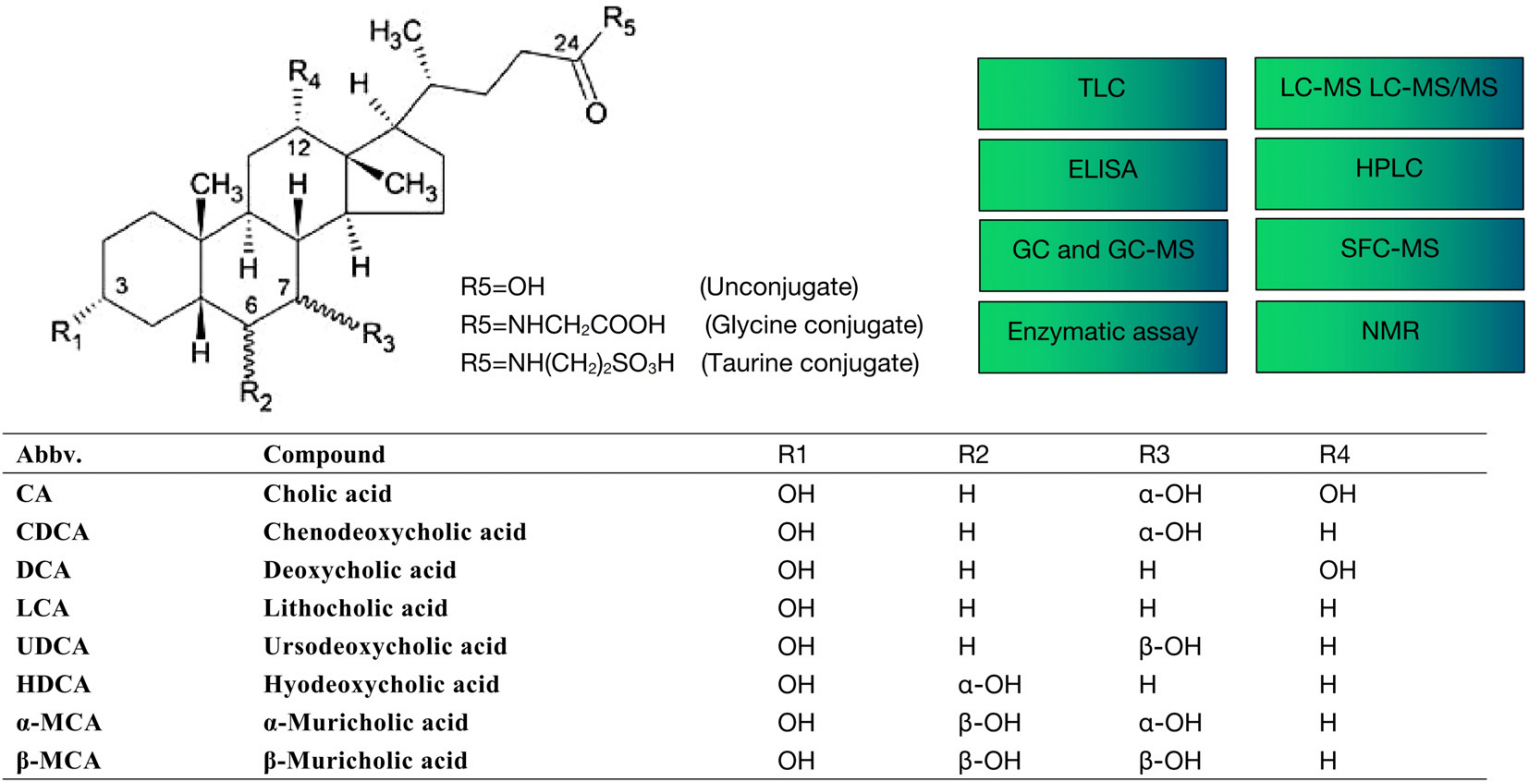
Structures of the most abundant bile acids found in humans and the various analytical platforms used to detect them.

Over the past decade, a variety of methodologies for bile acid (BA) separation, detection, and quantification have been reported. Initially, simpler yet robust techniques—such as enzymatic assays, enzyme-linked immunosorbent assays (ELISAs), thin-layer chromatography (TLC), high-performance liquid chromatography (HPLC), gas chromatography (GC), and supercritical fluid chromatography (SFC)—were widely used. More recently, the field has shifted toward high-throughput, highly sensitive methods, including GC–mass spectrometry (GC-MS), liquid chromatography–mass spectrometry (LC-MS), SFC–mass spectrometry (SFC-MS), and nuclear magnetic resonance (NMR) spectroscopy, which offer detailed molecular characterization and improved detection capabilities for BAs (9). More details can be seen in **Figure 2**.

Bile acids exert their biological effects primarily through two major receptors: the nuclear receptor FXR (NR1H4) and the membrane-bound G protein-coupled receptor TGR5 (GPBAR1). FXR exists in four distinct isoforms (FXRα1-4) with tissue-specific expression patterns, showing highest affinity for CDCA. Recent structural studies have revealed that the FXR ligand-binding domain undergoes specific conformational changes upon bile acid binding, leading to coactivator recruitment. TGR5, particularly abundant in skeletal muscle and brown adipose tissue, shows highest sensitivity to LCA and its conjugates. The tissue-specific distribution of these receptors is crucial for understanding their physiological roles, with both showing significant expression in metabolically active tissues (10, 11).

The signaling mechanisms of bile acids operate through multiple sophisticated pathways. FXR-mediated signaling involves both classical genomic pathways through direct DNA binding and transcriptional regulation, requiring the recruitment of coactivators such as PGC-1α and SRC-1. This nuclear receptor pathway regulates genes involved in bile acid, glucose, and lipid metabolism. Concurrently, TGR5-mediated signaling operates through cAMP-dependent pathways, leading to PKA-mediated CREB phosphorylation and subsequent enhancement of energy expenditure and glucose homeostasis. These signaling cascades demonstrate significant integration with other metabolic pathways, including insulin signaling, mitochondrial function regulation, and modulation of inflammatory responses (12, 13).

Recent research has revealed additional layers of complexity in bile acid signaling, particularly in their interaction with metabolic and inflammatory pathways. The cross-talk between bile acid signaling and various metabolic processes has emerged as a crucial regulatory mechanism in energy homeostasis and tissue function. This complex signaling network allows bile acids to function as sophisticated metabolic regulators, influencing not only energy homeostasis and glucose metabolism but also inflammatory responses. Of particular interest is their newly discovered role in muscle metabolism and function, suggesting significant therapeutic potential in metabolic disorders (14, 15).

## 3 Sarcopenia

### 3.1 Definition, pathophysiology and current diagnostic criteria

Sarcopenia, derived from the Greek words “sarx” (flesh) and “penia” (loss), represents a progressive and generalized skeletal muscle disorder characterized by accelerated loss of muscle mass, strength, and function (15). This age-related condition has evolved from its initial description to become recognized as a major geriatric syndrome with significant implications for public health. The European Working Group on Sarcopenia in Older People 2 (EWGSOP2) has recently updated its operational definition, emphasizing the central role of muscle strength as the primary parameter of sarcopenia (16).

The diagnosis of sarcopenia has been standardized through international consensus guidelines. According to EWGSOP2, low muscle strength (measured by grip strength or chair stand test) is the primary parameter for diagnosing probable sarcopenia. Confirmation requires documentation of low muscle quantity or quality, typically assessed through dual-energy X-ray absorptiometry (DXA) or bioelectrical impedance analysis (BIA) (17). Severe sarcopenia is diagnosed when poor physical performance, evaluated through gait speed, short physical performance battery, or timed-up-and-go test, accompanies low muscle strength and mass (18). The Asian Working Group for Sarcopenia (AWGS) has established population-specific cut-off points, recognizing ethnic variations in muscle mass and function (19).

### 3.2 Epidemiology and clinical significance

Sarcopenia is a multifactorial condition whose prevalence varies significantly across populations and diagnostic criteria, with recent meta-analyses indicating a range from 10% to 40% in adults aged 60 years and older (1). In long-term care settings, the rate can be even higher, and individuals over 80 face an elevated risk of up to 50% (20). Beyond the direct toll on physical function and independence, sarcopenia is associated with a host of adverse outcomes, such as increased likelihood of falls, fractures, hospitalization, and mortality.

Importantly, sarcopenia frequently coexists with a spectrum of metabolic derangements, including obesity, type 2 diabetes mellitus (T2DM), and non-alcoholic fatty liver disease (NAFLD). This clustering—often referred to as “sarcopenic obesity”—reflects a bidirectional relationship in which excessive adiposity and declining muscle mass exacerbate metabolic dysregulation (see Section 6.5). For example, patients with T2DM are more prone to accelerated muscle loss and diminished muscle quality, partly due to insulin resistance disrupting glucose uptake and protein synthesis in skeletal muscle (X). In parallel, chronic low-grade inflammation stemming from adipose tissue dysfunction can further impair muscle anabolism by elevating proinflammatory cytokine levels (IL-6, TNF-α), amplifying catabolic pathways (Y).

Growing evidence also suggests that declining muscle mass heightens vulnerability to broader metabolic syndrome, contributing to increased cardiometabolic risk and amplifying healthcare burdens. Sarcopenic individuals are more likely to experience reduced resting metabolic rate, decreased physical activity, and alterations in the gut–muscle axis—all of which further jeopardize metabolic health. These interdependencies underscore the importance of an integrated therapeutic approach that addresses both muscle preservation and metabolic control, rather than viewing sarcopenia in isolation.

Recognition of this synergy has prompted calls for diagnostic refinements and more holistic management strategies. Combining standard sarcopenia assessments (e.g., measuring grip strength and muscle mass) with evaluations of insulin sensitivity, liver function, and body composition may reveal clinically meaningful phenotypes that guide personalized interventions. From a public health perspective, targeting sarcopenia in conjunction with metabolic syndrome could significantly reduce the global burden of chronic diseases and the associated healthcare costs (21). Consequently, larger-scale prospective studies and multi-omics investigations are warranted to clarify the intricate mechanisms linking sarcopenia to broader metabolic dysfunction and to identify novel intervention points that can effectively address both conditions in tandem.

### 3.3 Molecular mechanisms of muscle wasting

The pathophysiology of sarcopenia involves complex interactions between multiple molecular pathways. Central to muscle wasting is the disruption of protein homeostasis, characterized by reduced protein synthesis and increased proteolysis (22). The ubiquitin-proteasome system and autophagy-lysosomal pathway are upregulated, while anabolic signaling through the IGF-1/Akt/mTOR pathway is diminished (23). Chronic inflammation, marked by elevated levels of pro-inflammatory cytokines (IL-6, TNF-α), contributes to muscle catabolism through NF-κB activation (24). Mitochondrial dysfunction plays a crucial role, leading to reduced ATP production, increased oxidative stress, and impaired muscle quality (25).

### 3.4 Risk factors and associated conditions

Multiple factors contribute to sarcopenia development, forming a complex network of interactions. Age-related hormonal changes, including decreased levels of testosterone, growth hormone, and IGF-1, significantly impact muscle maintenance (26). Physical inactivity, particularly sedentary behavior, accelerates muscle loss through mechanical unloading and metabolic derangements (27). Nutritional deficiencies, especially inadequate protein intake and vitamin D insufficiency, impair muscle protein synthesis and function (16). Chronic diseases, including diabetes, chronic obstructive pulmonary disease, and heart failure, exacerbate muscle wasting through various pathophysiological mechanisms (28).

### 3.5 Current therapeutic approaches

Management strategies for sarcopenia primarily focus on exercise interventions and nutritional optimization. Progressive resistance training remains the most effective intervention, stimulating muscle protein synthesis and improving muscle strength and function (29). Combined resistance and aerobic exercise programs show particular promise in addressing both muscle and functional deficits (30). Nutritional interventions, including adequate protein intake (1.0-1.2 g/kg/day) and vitamin D supplementation, support muscle maintenance and function (31). Emerging pharmacological approaches target specific pathways involved in muscle wasting, including myostatin inhibitors, selective androgen receptor modulators (SARMs), and ghrelin agonists, though their long-term efficacy and safety require further investigation (32).

## 4 Dysregulation of bile acid metabolism in sarcopenia

**Figure 3:**
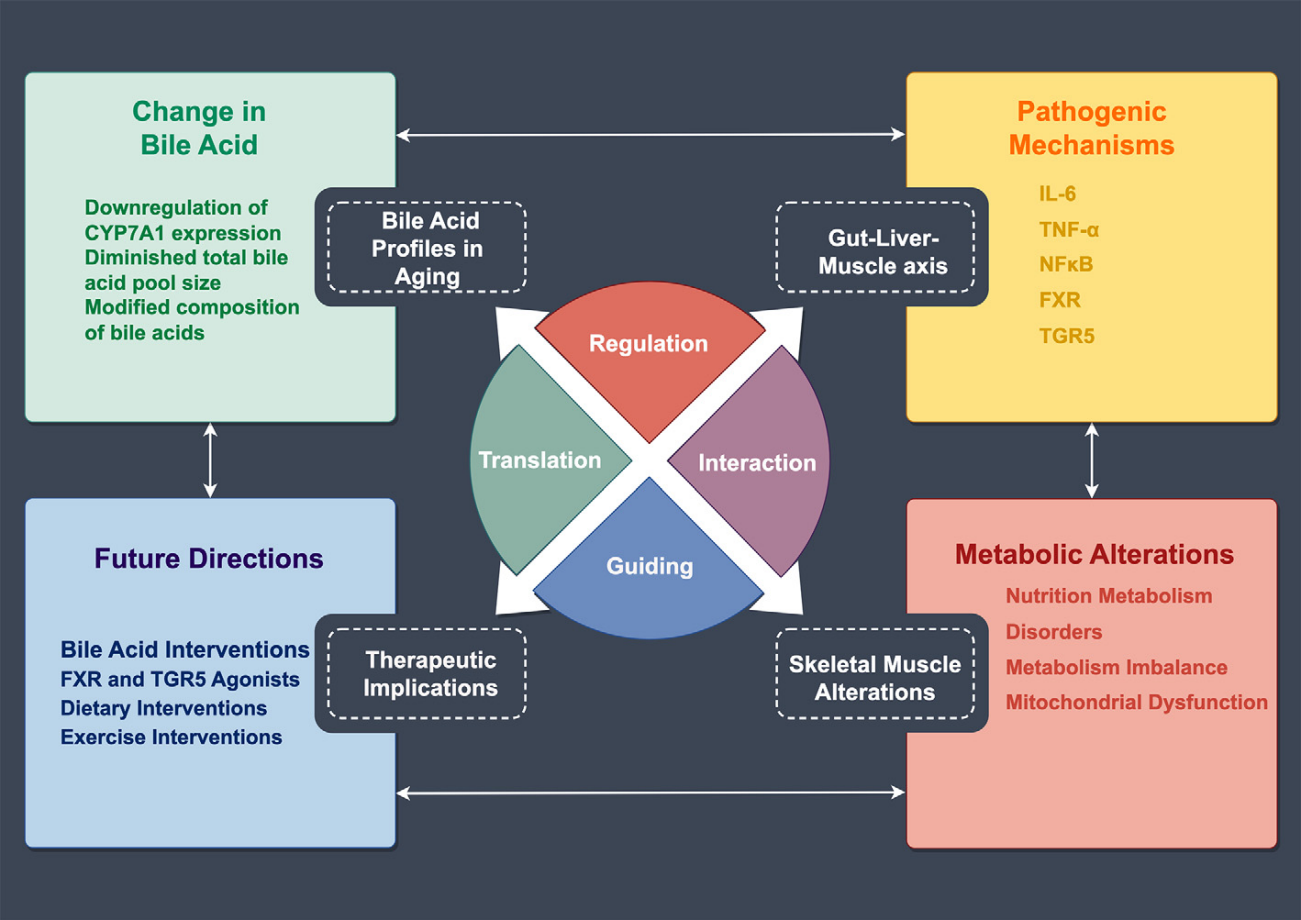
Regulatory Network of Bile Acid Metabolism and Skeletal Muscle Changes in Aging. This schematic illustrates the mechanistic relationships between age-related bile acid metabolism and skeletal muscle alterations. The central circular diagram comprises four regulatory modules: Regulation, Translation, Interaction, and Guiding. The upper left panel shows changes in bile acids, including CYP7A1 downregulation, reduced bile acid pool size, and modified composition. The upper right panel lists pathogenic mechanisms involving inflammatory factors (IL-6, TNF-α) and receptors (FXR, TGR5). The lower right panel demonstrates metabolic alterations, encompassing nutrition metabolism, metabolic disorders, and mitochondrial dysfunction. The lower left panel outlines future directions, including bile acid interventions, receptor agonists, and dietary and exercise interventions.

The intricate relationship between bile acid metabolism and skeletal muscle function has emerged as a crucial area of investigation in understanding sarcopenia pathophysiology. Recent evidence suggests that age-related alterations in bile acid homeostasis may contribute significantly to muscle deterioration, establishing a novel perspective on the metabolic aspects of sarcopenia (33) (**Figure 3**).

### 4.1 Alterations in bile acid profiles in aging

Aging is associated with substantial changes in bile acid metabolism and composition. Studies have demonstrated that elderly individuals exhibit distinct bile acid profiles characterized by reduced total bile acid pool size and altered composition of primary and secondary bile acids (34). Specifically, aging leads to decreased synthesis of primary bile acids through downregulation of CYP7A1 expression, the rate-limiting enzyme in bile acid synthesis (3). Moreover, the ratio of hydrophobic to hydrophilic bile acids increases with age, potentially contributing to cellular stress and inflammatory responses in various tissues, including skeletal muscle.A study (35)showed that UDCA, CDCA, TCA, TUDCA, TCDCA, GUDCA, GCDCA, and GCA were elevated, suggesting that TUDCA, TCDCA, and GCDCA are associated with liver function and may serve as predictive markers for cirrhosis. Recent metabolomic analyses have revealed significant correlations between specific bile acid species and muscle mass indices, suggesting their potential role as biomarkers for sarcopenia (36).

### 4.2 Changes in bile acid signaling in sarcopenic patients

The dysregulation of bile acid signaling pathways represents a critical mechanism linking altered bile acid metabolism to muscle wasting in sarcopenia. Farnesoid X Receptor (FXR) and Takeda G-protein-coupled receptor 5 (TGR5) expression patterns show significant alterations in skeletal muscle of sarcopenic patients (8). These changes affect downstream signaling cascades crucial for muscle protein synthesis and energy metabolism. Notably, reduced TGR5 signaling in aged muscle has been associated with decreased mitochondrial function and impaired glucose uptake (37). Furthermore, alterations in FXR-mediated pathways affect muscle protein turnover through modulation of mTOR signaling and autophagy regulation (38).

### 4.3 Impact of liver dysfunction on muscle wasting

The liver-muscle axis plays a fundamental role in the pathogenesis of sarcopenia through its effects on bile acid metabolism. Age-related hepatic dysfunction leads to impaired bile acid synthesis and processing, contributing to systemic metabolic perturbations (39). Clinical studies have demonstrated that patients with liver cirrhosis exhibit accelerated muscle loss, partially attributed to disrupted bile acid signaling (40). The compromised enterohepatic circulation of bile acids in elderly individuals further exacerbates metabolic dysfunction in skeletal muscle, creating a vicious cycle of progressive muscle deterioration (9).

### 4.4 Gut microbiota, bile acids, and muscle health

The gut microbiota emerges as a critical mediator in the relationship between bile acid metabolism and muscle health. Age-related changes in gut microbiota composition significantly affect bile acid transformation and signaling (41). Reduced diversity and altered abundance of bile acid-metabolizing bacteria in elderly individuals lead to modified secondary bile acid profiles, affecting muscle metabolism and function (11). Recent studies have demonstrated that specific microbial metabolites derived from bile acid transformation can influence muscle mass and strength through various signaling pathways (42). The gut-liver-muscle axis represents a complex network where perturbations in microbial bile acid metabolism contribute to sarcopenia development (43).

### 4.5 Inflammatory mediators and their role

Chronic inflammation, a hallmark of aging and sarcopenia, significantly influences bile acid metabolism and its effects on muscle health. Pro-inflammatory cytokines, particularly IL-6 and TNF-α, modulate bile acid synthesis and signaling pathways (44). These inflammatory mediators can disrupt normal bile acid homeostasis, leading to altered muscle metabolism and protein turnover (45). Additionally, bile acids themselves can influence inflammatory responses in muscle tissue through FXR-dependent regulation of NF-κB signaling (46). The complex interplay between inflammatory mediators and bile acid signaling creates a regulatory network that significantly impacts muscle mass maintenance (47).

Recent research has identified several therapeutic implications of targeting bile acid metabolism in sarcopenia. Interventions aimed at normalizing bile acid profiles or enhancing bile acid signaling show promise in improving muscle health (48). These include the use of bile acid receptor agonists, probiotics targeting bile acid-metabolizing bacteria, and strategies to optimize the gut-liver-muscle axis (49). Understanding the molecular mechanisms underlying bile acid dysregulation in sarcopenia opens new avenues for therapeutic intervention in age-related muscle wasting (50).

### 4.6 Harnessing multi-omics integration to decode bile acid signaling in muscle health and disease

Multi-omics integration—including genomics, epigenomics, transcriptomics, proteomics, metabolomics, and microbiomics—offers a powerful framework for deepening our understanding of bile acid signaling in skeletal muscle. While single-omics analyses have successfully identified individual biomolecules associated with muscle health, a multi-omics approach can uncover complex interactions and regulatory networks that drive muscle homeostasis and disease progression. For instance, coupling transcriptomic data on gene expression changes in bile acid receptors (e.g., FXR, TGR5) with untargeted metabolomic profiling could pinpoint precise metabolic pathways affected in sarcopenia. Parallel proteomic and microbiomic analyses may further elucidate how the gut-muscle axis modulates bile acid composition, thereby shaping clinical outcomes.

Through advanced computational methods and machine learning, multi-omics datasets can be integrated to generate predictive models of disease risk, treatment response, and baseline ‘omics signatures’ indicative of distinct muscle phenotypes. This holistic view may reveal novel biomarker candidates—such as unique bile acid derivatives or receptor cofactors—capable of improving early detection or stratifying therapeutic interventions. Moreover, it can inform the rational design of targeted compounds that modulate specific aspects of bile acid signaling pathways, increasing the likelihood of therapeutic success and minimizing off-target effects. By adopting multi-omics strategies, future studies can accelerate translation from bench to bedside, laying the groundwork for personalized interventions in sarcopenia and other muscle-related conditions.

## 5 Clinical evidence

### 5.1 Human studies linking bile acid metabolism and muscle mass

A growing body of clinical evidence establishes a compelling connection between bile acid metabolism and muscle health in diverse human populations. In large-scale cohort studies of older adults, serum bile acid profiles—particularly alterations in deoxycholic acid and chenodeoxycholic acid—were strongly correlated with decreased appendicular skeletal muscle mass index (48, 51). These findings suggest that quantifying specific bile acid derivatives could offer a useful biomarker panel for early identification of individuals at risk of muscle wasting. Research has revealed that cirrhosis patients with sarcopenia experience higher levels of secondary bile acids (such as LCA, GLCA, and GDCA) in their stool, accompanied by lower serum concentrations of valine and alanine. Similarly, patients with nonalcoholic fatty liver disease (NAFLD) exhibit elevated plasma bile acid levels, which show an inverse correlation with skeletal muscle volume. Among the various bile acid subtypes, DCA is the most notably dysregulated in NAFLD patients. Due to its role in promoting protein catabolism and energy expenditure via TGR5 activation, DCA has been proposed as a promising biomarker for sarcopenia associated with NAFLD (45).

While high-level clinical trials are still lacking, some evidence suggests that bile acids may improve muscle strength and function. However, these findings are primarily based on studies with short durations and relatively small sample sizes. TGR5 inhibitors present a potential alternative for reducing muscle mass loss in patients with CLD and warrant further investigation. One notable example is SBI-115, a TGR5 inhibitor that has shown promising results in preclinical models of other liver diseases.Additionally, preliminary research using synthetic bile acid analogues has demonstrated enhanced muscle protein synthesis and reduced inflammatory markers, further supporting the potential mechanistic role of bile acids in regulating both anabolic and catabolic pathways.

### 5.2 Animal models and experimental evidence

Preclinical studies have provided mechanistic insights into the role of bile acids in muscle metabolism. Mouse models with genetic modifications in bile acid signaling pathways exhibit accelerated muscle wasting and impaired regeneration capacity (46). Notably, FXR knockout mice demonstrate reduced muscle mass and strength, accompanied by altered protein synthesis and degradation pathways (47). Experimental evidence using targeted metabolomics has revealed that bile acid supplementation can enhance muscle protein synthesis through activation of mTOR signaling in aged rodents (48).

### 5.3 Biomarkers and diagnostic implications

The identification of bile acid-related biomarkers has emerged as a promising approach for early sarcopenia detection. Longitudinal studies have shown that specific bile acid metabolites in serum can predict muscle mass decline with high sensitivity and specificity (49). Integration of bile acid profiles with conventional sarcopenia markers has improved diagnostic accuracy, particularly in identifying pre-sarcopenic states (50). Recent metabolomic analyses have established a panel of bile acid species that correlate strongly with muscle function parameters (51).

### 5.4 Gender-specific differences

Clinical observations have revealed significant gender-specific variations in bile acid metabolism and its relationship with muscle mass. Women typically exhibit different bile acid profiles compared to men, with distinct associations to muscle strength and function (48). Post-menopausal women show particularly pronounced alterations in bile acid homeostasis, correlating with accelerated muscle loss (52). These differences may be attributed to hormonal influences on bile acid synthesis and signaling pathways (53).

### 5.5 Age-related variations

Age-dependent changes in bile acid metabolism significantly impact muscle health across different life stages. Longitudinal studies have documented progressive alterations in bile acid composition with advancing age, correlating with declining muscle mass (53). The elderly population demonstrates reduced bile acid synthesis and modified enterohepatic circulation, potentially contributing to age-related muscle wasting (54).

## 6 Therapeutic implications

### 6.1 Bile acid-based interventions

Emerging therapeutic strategies targeting bile acid metabolism show promise in preventing and treating sarcopenia. Clinical trials investigating bile acid supplementation have reported improvements in muscle mass and function in elderly participants (55). Synthetic bile acid analogues have demonstrated potential in enhancing muscle protein synthesis and reducing inflammation in preliminary studies (47).

### 6.2 FXR and TGR5 agonists in muscle preservation

Pharmacological activation of bile acid receptors represents a novel therapeutic approach. Selective FXR agonists have shown efficacy in preserving muscle mass in both preclinical models and early clinical trials (56). Similarly, TGR5 activation through specific agonists has demonstrated beneficial effects on muscle metabolism and function (46). Combined targeting of these receptors may offer synergistic benefits in muscle preservation (57).

### 6.3 Dietary interventions affecting bile acid metabolism

Nutritional strategies modulating bile acid metabolism have emerged as practical interventions. High-fiber diets and specific nutrient combinations can favorably alter bile acid profiles and improve muscle health (58). Dietary supplements targeting bile acid synthesis and signaling pathways have shown promising results in maintaining muscle mass (59). Therapeutic approaches targeting the gut microbiota have demonstrated potential in optimizing bile acid metabolism and muscle function. Probiotic interventions specifically designed to enhance bile acid transformation have shown positive effects on muscle mass maintenance (60). Prebiotic supplementation strategies aimed at modifying the gut microbiome composition have demonstrated improvements in bile acid profiles and muscle strength (61).

### 6.4 Exercise and bile acid homeostasis

Physical activity significantly influences bile acid metabolism and its effects on muscle health. Regular exercise has been shown to normalize bile acid profiles and enhance metabolic signaling (62). Combined exercise and bile acid-targeted interventions demonstrate promising outcomes in metabolic health (63).

### 6.5 Challenges in translating bile acid signaling to clinical practice

Despite the promising therapeutic potential of bile acid-mediated pathways in skeletal muscle disorders, several key challenges remain in the path toward clinical translation. These include the heterogeneity in bile acid profiling methodologies across different laboratories, the intricate interplay of gut microbiota, and the need for large-scale, randomized controlled trials to establish efficacy and safety. Additionally, the complexity of individualized patient factors—ranging from genetic polymorphisms to comorbid conditions—further underscores the necessity for personalized interventions. Addressing these challenges necessitates a concerted cross-disciplinary effort, harmonized protocols for bile acid quantification, and advanced multi-omics technologies to facilitate a deeper understanding of bile acid-muscle crosstalk under physiological and pathological conditions.

## 7 Limitations

Despite significant advances in bile acid research, several critical knowledge gaps persist. The temporal relationship between bile acid alterations and muscle mass decline requires further longitudinal investigation (57), while the complex interplay between bile acid signaling and muscle homeostasis pathways remains incompletely understood (64). High-throughput metabolomics platforms now enable comprehensive bile acid profiling, potentially leading to early detection of muscle-related disorders. The integration of artificial intelligence with bile acid biomarker analysis shows promise in improving diagnostic accuracy (65), while novel imaging techniques combined with metabolomics may offer more precise assessment of muscle quality. Additionally, the role of specific bile acid species in muscle protein synthesis and degradation necessitates more detailed exploration.

## 8 Conclusion

The translation of basic research findings into clinical applications presents both challenges and opportunities. While the modulation of bile acid receptors, particularly FXR and TGR5, represents a promising intervention strategy (66), the complexity of bile acid signaling networks presents challenges in developing targeted therapies. Personalized therapeutic approaches based on individual bile acid profiles are being developed, and novel drug delivery systems targeting specific bile acid pathways show potential for enhanced efficacy. The gut microbiota-bile acid axis has emerged as another therapeutic target, with innovative approaches focusing on microbiome modulation (67). However, standardization of bile acid measurement techniques across laboratories remains crucial. The integration of multi-omics approaches may provide new insights into bile acid-muscle interactions (68), potentially leading to more effective therapeutic strategies for muscle-related disorders.
